# Genetic profiling of poorly differentiated sinonasal tumours

**DOI:** 10.1038/s41598-018-21690-6

**Published:** 2018-03-05

**Authors:** Alejandro López-Hernández, Blanca Vivanco, Alessandro Franchi, Elisabeth Bloemena, Virginia N. Cabal, Sira Potes, Cristina Riobello, Cristina García-Inclán, Fernando López, José L. Llorente, Mario Hermsen

**Affiliations:** 1 Department Otolaryngology, IUOPA, CIBERONC, Hospital Universitario Central de Asturias, Oviedo, Asturias Spain; 2 Department Pathology, Hospital Universitario Central de Asturias, Oviedo, Spain; 3 Department of Translational Research and of New Technologies in Medicine and Surgery, University of Pisa, Pisa, Italy; 4 Department Pathology, VUmc, Amsterdam, The Netherlands

## Abstract

The sinonasal cavities harbour a variety of rare tumour types. Many carry a poor prognosis while therapeutic options are limited. Histopathological classification can be difficult, especially for poorly differentiated tumours such as olfactory neuroblastoma (ONB), sinonasal neuroendocrine carcinoma (SNEC) and sinonasal undifferentiated carcinoma (SNUC). We analysed Affymetrix OncoScan genome-wide copy number profiles of these three tumour types, both as originally diagnosed and as regrouped by their cytokeratin (Ck) and neuroendocrine (Ne) expression pattern, aiming to find a relation between phenotype and genotype. According to the original histopathological classification our series consisted of 24 ONB, 11 SNEC and 19 SNUC, while immunohistochemistry indicated 11 Ck−Ne+/ONB, 18 Ck+Ne+/SNEC, 24 Ck+Ne−/SNUC, and 1 Ck−Ne−/unclassified. As originally diagnosed, the three tumour types showed similar copy number profiles. However, when regrouped by Ck/Ne immunostaining we found a distinct set of gains and losses; Ck−Ne+/ONB harboured few and predominantly whole chromosomes abnormalities, Ck+Ne+/SNEC carried both gains and losses in high frequency, and Ck+Ne−/SNUC showed mostly gains. In addition, each tumour carried a number of unique chromosomal deletions. Genome-wide copy number profiling supports the value of immunohistochemical CkNe staining of ONB, SNEC and SNUC for tumour classification, which is important for prognosis and therapeutic decision-making.

## Introduction

The sinonasal cavities are an anatomical area from which emerges a wide histological diversity of neoplasms^1^. Malignant sinonasal tract tumours represent approximately 5% of all head and neck neoplasms and have distinctive etiology, epidemiology, clinical and genetic characteristics^2^. Sinonasal squamous-cell carcinoma (SNSCC) and intestinal-type adenocarcinoma (ITAC) comprise roughly 70% of sinonasal cancers, while the remaining 30% include a miscellany of neuroendocrine carcinomas, neuroectodermal neoplasms, salivary gland tumours and undifferentiated carcinomas^3^. Diagnosis based primarily on morphological and histological features is a challenge for the pathologist, especially for those tumours with poor differentiation such as olfactory neuroblastoma (ONB), sinonasal neuroendocrine carcinoma (SNEC) and sinonasal undifferentiated carcinoma (SNUC). Moreover, as these tumours have an incidence of approximately 1/100.000 inhabitants, it is hard to acquire sufficient experience. ONB and SNEC can appear very similar and may also be mistaken for SNUC^4^. Indeed, in the past SNUCs were included in the group of high-grade ONB or remained unclassified for absence of specific criteria. The 2017 World Health Organization (WHO) classification of head and neck tumours has defined SNUC as a highly aggressive tumour of uncertain histogenesis without evidence of squamous or glandular differentiation^1^ thus representing a category with a diagnosis of exclusion. Some evidence for neuroendocrine differentiation in SNUC has been reported^5,6^, but most authors do not classify them as neuroendocrine neoplasms for the lack of neuroendocrine immunoreactivity^7–9^.

Several immunohistochemical markers, including pan-cytokeratin, synaptophysin, chromogranin, neuron-specific enolase, CD56, desmin, S-100 and melanin, have been suggested to overcome the classification difficulties of poorly differentiated sinonasal tumours^4,7,10–14^. In addition, some tumours can now be identified by molecular genetic analysis^15–17^, for example NUT (NUclear protein in Testis) carcinoma and SMARCB1 (INI-1) deficient sinonasal carcinoma^18,19^. Until the present moment no specific diagnostic molecular markers are known for ONB, SNEC and SNUC.

Correct diagnosis is important because of its relation to clinical outcome^20–23^. Generally SNEC, SNUC, and NUT carcinoma have the worst prognosis with a survival of 9–18 months, in spite of aggressive treatment^1,8,23^. In a study of 23 head and neck neuroendocrine carcinomas including 11 cases occurring in the sinonasal cavities (i.e. SNEC), the 5-year overall survival was 91.5% for well and moderately differentiated tumours, dropping to 21.1% for poorly differentiated cases^13^. In ONB, 5-year overall survival may go down from 80% to 40% in low grade and high grade tumours, respectively^1,8,23^. All sinonasal tumour patients frequently develop local recurrences (50–80%) which are the principal cause of death. Lymph node or distant metastasis are relatively rare, occurring in approximately 10–20% of cases^1,2,8^.

Correct tumour classification is also relevant for therapeutic decision-making. Currently, the optimal treatment for ONB is surgery combined with radiotherapy, while SNUC and SNEC may receive multimodality treatment with chemotherapy^4,20,22^. With the development of novel inhibitors targeted to specific genetic alterations, personalized therapeutic opportunities are becoming available also for sinonasal cancer patients.

In this study we aimed to find a relation between the phenotype and genotype of 54 ONB, SNEC and SNUC, using Affymetrix OncoScan genome-wide copy number analysis. We compared the tumours grouped according to the original histopathological classification and according to their cytokeratin (Ck) and neuroendocrine (Ne) marker expression pattern: ONB was defined as Ck−Ne+, SNEC as Ck+Ne+ and SNUC as Ck+Ne−.

## Results

### Immunohistochemical classification

**Figure 1:**
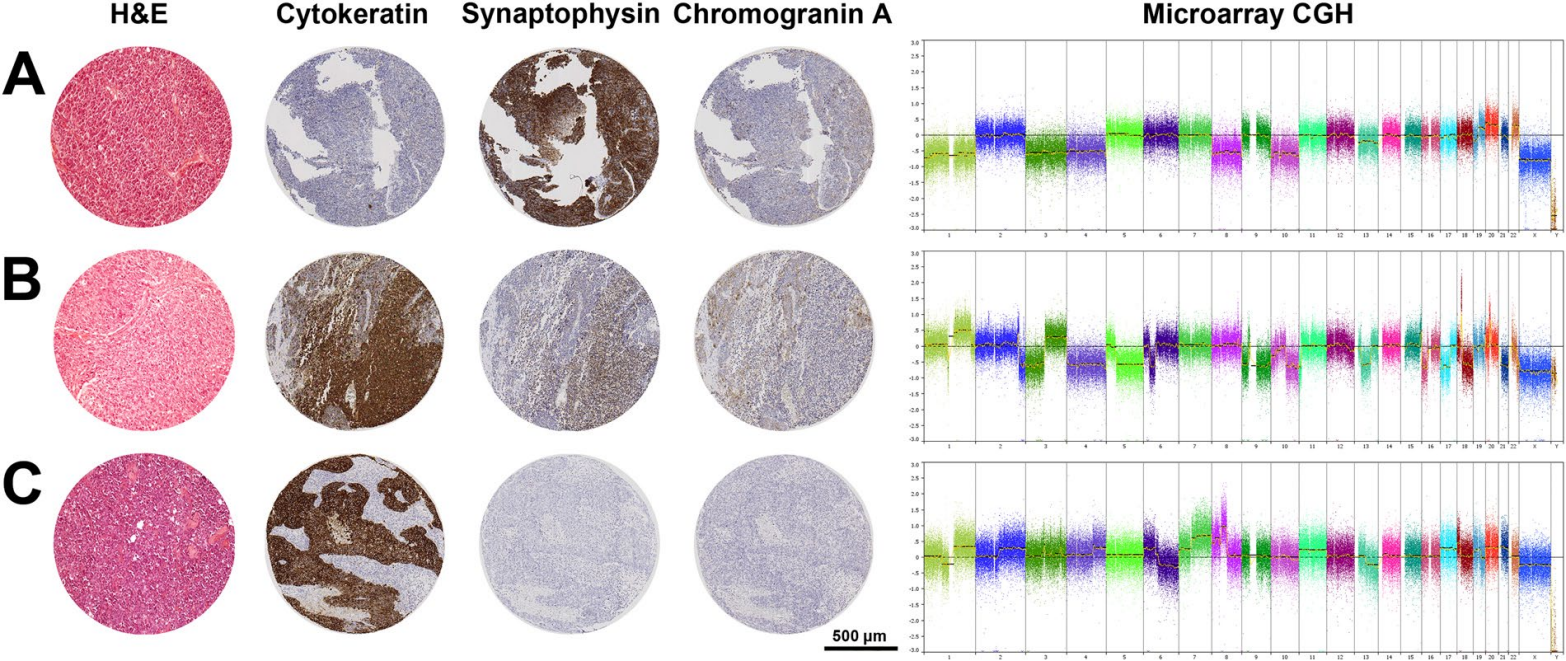
Representative cases of (**A**) Ck−Ne+/ONB, (**B**) Ck+Ne+ and (**C**) Ck+Ne−/SNUC. Shown are H&E staining, cytokeratin, synaptophysin and cromogranin A immunostainings, and genome-wide copy number analysis. The Ck−Ne+/ONB case (**A**) shows few aberrations, mainly involving whole chromosomes. The Ck+Ne+/SNEC case (**B**) carries many aberrations affecting whole chromosomes, chromosome arms chromosome segments, and high level amplifications. The Ck+Ne−/SNUC case (**C**) harbours many aberrations affecting whole chromosomes, chromosome arms chromosome segments.

Staining of 54 sinonasal tumours with Ck and Ne markers (Fig. 1) indicated 11 cases as Ck−Ne+/ONB, all of which orginally diagnosed as ONB. Ck+Ne+/SNEC staining was observed in 18 cases, of which 9 had orginally been evaluated as ONB, 8 as SNEC and 1 as SNUC. A Ck+Ne−/SNUC profile was seen in 24 cases, and of these, 4 had orginally been classified as ONB, 2 as SNEC and 18 as SNUC. One case orginally classified as SNEC was negative for both markers, Ck−Ne− (Table 1). In total 17 tumours were differently diagnosed comparing the original and immunohistochemical CkNe classification. Immunohistochemical staining for SMARCB1 revealed loss of expression in two Ck+Ne−/SNUC cases and one other Ck+Ne−/SNUC case showed focal NUT-1 staining in 30–35% of cells, which was below the threshold of positivity.

**Table 1:** Original diagnosis and IHC classification of 54 sinonasal tumours.

Initial diagnosis			IHC classification			
Tumour entity	Differentiation	N° of Cases	Ck−Ne+	Ck+Ne+	Ck+Ne−	Ck−Ne−
ONB	Grade I	3	3			
	Grade II	10	5	2	3^a^	
	Grade III	6	1	5		
	Grade IV	5	2	2	1	
SNEC		11		8	2	1
SNUC		19		1	18^b,c^	
Total		24	11	18	24	1

Legend. ONB: olfactory neuroblastoma; SNEC: sinonasal neuroendocrine carcinoma; SNUC sinonasal undifferentiated carcinoma; Ck: pan-cytokeratin marker (AE1/AE3); Ne: neuroendocrine markers (synaptophysin/cromogranin); ^a^one case is also SMARCB1-deficient; ^b^one case is also SMARCB1-deficient; ^c^one case shows focal NUT staining.

### Genomic profiling

**Figure 2:**
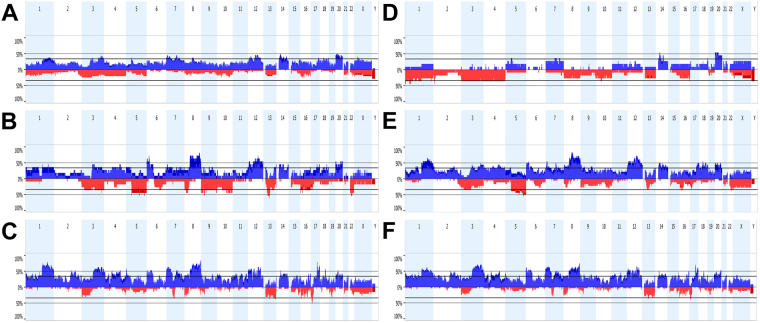
Copy number aberration profiles of 24 ONB (**A**), 11 SNEC (**B**) and 19 SNUC (**C**) according to the original diagnosis, and 11 Ck−Ne+/ONB (**D**), 18+Ne+/SNEC (**E**) and 24 Ck+Ne−/SNUC (**F**) according to the immunohistochemical reclassification. The percentage of cases with copy number gains and losses are given in blue and red, respectively.

Genome-wide copy number analysis revealed alterations in all 54 tumours. The majority had complex karyotypes with gains and losses involving many chromosomes, but a number of cases carried few aberrations, mostly being whole chromosome gains or losses (Fig. 1). Overall, the three tumour types originally diagnosed showed CNAs involving most chromosomes, but with differences in frequency. Considering the chromosomal regions with >35% CNAs, in ONB we observed gains at 1q, 3q, 7, 8q, 12, 14 and 20, in SNEC gains at 1q, 3q, 4q, 6p, 8q, 12q and 20q, and losses at 3, 5q, 8p, 9, 10, 13, 16 and 22, and in SNUC gains at 1q, 2q, 3q, 4q, 6p, 7, 8q, 12, 17q and 20, and losses at 13q and 17p (Fig. 2A–C).

The genomic profiles of the same tumours regrouped according to the Ck and Ne staining demonstrated a better defined map of CNA hotspots with higher frequencies within each new group (Fig. 2D–F). The Ck−Ne+/ONB cases differed considerably from the original ONB and showed a markedly low frequency of CNAs, with gains in >35% of cases at 7q, 14q, 18q and 20 and losses at 1p, 2p, 3 and 4 (Table 2). Losses were more frequent than gains. Eight of 11 cases carried CNAs concerning whole chromosomes, whereas CNAs of segments of chromosomes were rare (Fig. 1). Ck+Ne+/SNEC tumours showed a profile with frequent gains and losses. Hotspot gains of >50% were seen at 1q, 6p, 7, 8q, 12, 14, 17q, 18q and 20, and >35% losses occurred at 5q, 16p and 22q (Table 2). Compared to the original SNEC profile, hotspot gains remained similar, whereas losses at 3q, 9, 10 and 13q became less pronounced. The recurrent aberrations in the Ck+Ne−/SNUC cases did not differ much from the original SNUC profile because the immunohistochemical reclassification changed only few cases; many gains occurred in >50% of cases, while losses were infrequent, only at 13q and 17p in >35% of cases. One case was Ck−Ne-/unclassified and showed mainly whole chromosome CNAs, similar to the Ck−Ne+/ONB tumours.

**Table 2:** Frequencies of chromosomal copy number alterations of sinonasal tumours according to the IHC profile.

Hotspots	Alteration	Ck−Ne+/ONB (n = 11)	Ck+Ne+/SNEC (n = 18)	Ck+Ne−/SNUC (n = 24)
1p	Loss	27–45%	6–11%	0–8%
2p25.1-14.3	Loss	9–45%	0–6%	0–12%
3	Loss	36%	22–33%	0–25%
4	Loss	36%	6–17%	0–21%
5q	Loss	9%	39–56%	4–17%
13q14.3-q21.1	Loss	27%	28–33%	25–38%
16p12.3	Loss	18–27%	28–39%	17–21%
17p13.1-p11.2	Loss	0	17–33%	21–42%
22q12.2-12.3	Loss	9–18%	28–39%	13–21%
1q	Gain	0–18%	39–67%	46–66%
2p25-13	Gain	0%	11–39%	25–42%
2q23.3-34	Gain	0%	22–39%	33–50%
3q	Gain	0–18%	28–44%	29–71%
4p16	Gain	0	28–39%	29–50%
4p14-13	Gain	0–9%	28–33%	17–46%
4q13-31	Gain	0–18%	28–44%	21–50%
6p	Gain	0–9%	33–50%	25–63%
7p	Gain	18–27%	22–56%	25–67%
7q11.23-33	Gain	18–36%	22–50%	29–50%
8p	Gain	0	33–44%	33–38%
8q	Gain	0%	56–83%	45–75%
9p24	Gain	0–9%	39–44%	13–42%
9q22.1-31.1	Gain	0	33–39%	25–42%
11p14.3-p12	Gain	9–18%	11–28%	21–42%
11q13.4-q14.2	Gain	9–18%	11–28%	21–46%
12p	Gain	9–27%	44–56%	38–54%
12q	Gain	9–27%	44–72%	25–67%
14q	Gain	18–55%	28–50%	25–42%
16q23.3-24.3	Gain	9–18%	11–22%	38–50%
17q22-25	Gain	0–18%	28–50%	29–71%
18q	Gain	18–36%	44–72%	17–50%
20	Gain	18–55%	28–50%	17–54%

In contrast to the original classification, the three reclassified tumours showed more pronounced differences in their CNA profiles. Ck−Ne+/ONB showed few changes and mostly losses, Ck+Ne+/SNEC had both gains and losses in high frequency, and Ck+Ne−/SNUC harboured mostly gains. Especially the Ck−Ne+/ONB group stood out by having relatively simple karyotypes with mainly gains or losses of whole chromosomes. The absence of CNAs of segments of chromosomes, indicating breakage and chromosomal instability, was in stark contrast with the high frequency found in Ck+Ne+/SNEC and Ck+Ne−/SNUC.

A number of chromosomal losses appeared almost exclusively in one of the three tumours: 1p, 2p and 4 in Ck−Ne+/ONB, 5q, 6q, 9q, 16p and 22q in Ck+Ne+/SNEC, and 7q and 17p in Ck+Ne−/SNUC. Many gains occurred in all three tumours, although gains at 18q were particularly frequent in Ck+Ne+/SNEC, and gains at 3q, 7p, 11, 16q and 17q especially frequent in Ck+Ne−/SNUC. A notable absence of gains at 8q was observed in Ck−Ne+/ONB (Fig. 2). Table 2 lists 22 hotspot regions showing recurrent gains and losses in >35% in at least one of the tumour types.

**Figure 3:**
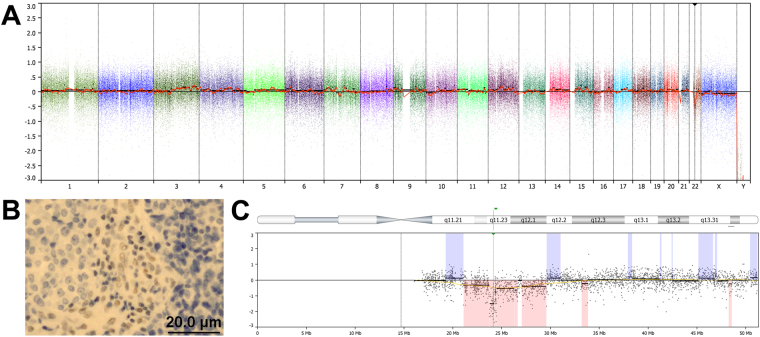
SMARCB1-deficient Ck+Ne−/SNUC case. Genome-wide copy number analysis showed a simple karyotype, with only a few CNAs, including gain at 3q, focal gains at 1p, 7p and 7q and a loss at 22q (**A**). Immunohistochemical analysis with antibody anti-INI-1 showed absence of SMARCB1 expression, while infiltrating lymphocytes were positive (**B**). Detail of the deletion at 22q: right at the site where the gene SMARCB1 is localized (arrow), the deletion is homozygous (**C**).

**Figure 4:**
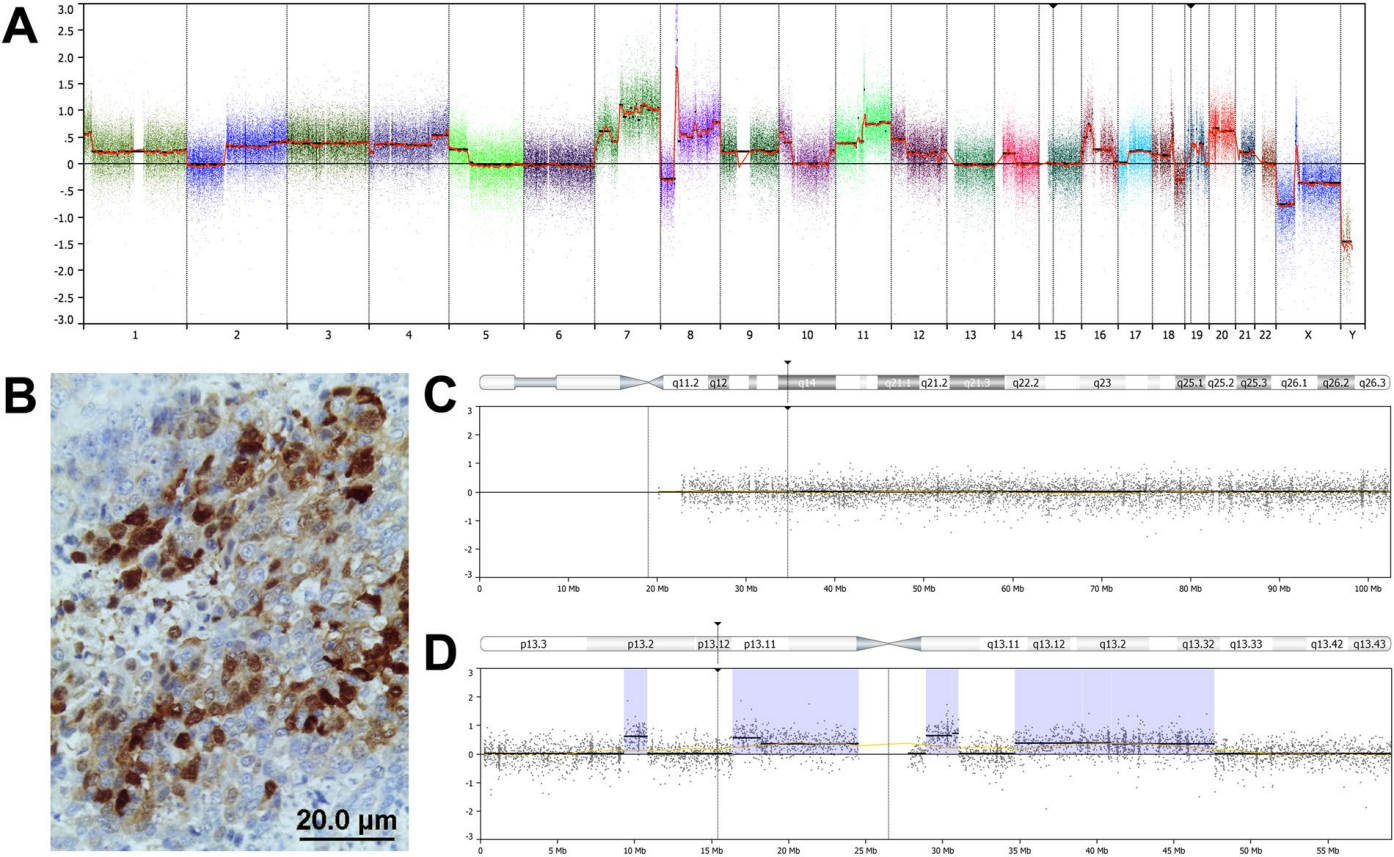
Ck+Ne−/SNUC case as suspected NUT carcinoma. Genome-wide copy number analysis showed a complex karyotype with losses, gains and amplifications (**A**). Immunohistochemical analysis with antibody anti-NUT-1 showed a focal staining in 30–35% of tumour cells (**B**). Details of chromosome 15 (**C**) on chromosome 19 (**D**): at the localizations of the genes NUT-1 and BRD4 (arrows) no aberrations are present.

Within the Ck+Ne−/SNUC group, two tumours were found SMARCB1-deficient and one showed NUT-1 staining in 30–35% of tumour cells. The two SMARCB1 deficient cases had few CNAs, one carried a homozygous deletion at 22q11, where the SMARCB1 gene is localized (Fig. 3). The case with focal NUT-1 staining showed a complex karyotype with many gains and losses, however, no gains or losses were observed at the NUT-BRD4 translocation breakpoints on chromosomes 15 and 19 (Fig. 4).

## Discussion

Poorly differentiated sinonasal tumours can be hard to diagnose correctly. In this study we collected specimens of ONB, SNEC and SNUC from three different hospitals and we regrouped this set of tumours on the basis of their Ck and Ne immunoprofile, which changed 17 of 54 tumours to another category. Surprisingly, these cases were not only high grade tumours; 5 ONBs with grade II differentiation were reclassified to 2 Ck+Ne+/SNEC and 3 Ck+Ne−/SNUC (Table 1). We found that the immunohistochemical CkNe grouping resulted in groups of tumours with better defined copy number alteration profiles compared to the original classification.

Ck−Ne+/ONB harboured few abnormalities involving mostly whole chromosome gains and losses. Whole chromosome CNAs have been reported previously in ONB^24,25^. In our study, 8 of 11 Ck−Ne+/ONB were grade I and II, therefore it may be that whole chromosome aberrations and a low level of chromosomal instability are characteristic for low-grade Ck−Ne+/ONB. Ck+Ne+/SNEC and most Ck+Ne−/SNUC on the other hand, revealed many CNAs affecting chromosome segments, chromosome arms and whole chromosomes, thus reflecting a high level of chromosomal instability. Apart from these genome-wide differences, the reclassified tumours also harboured distinct gains and losses at specific chromosomal regions, although also common alterations were observed, for example gains at chromosomes 7, 14 and 20, and losses at 13q and 16p.

Only few whole genome copy number studies have been published on ONB, SNEC or SNUC to compare our data with. The findings of Bockmuhl *et al*.^24^ on ONB, with frequent losses at 1p, 3p, 9p, and 10, and gains at 20, are similar to our data. Two other ONB studies, however, obtained a different CNA profile^25,26^. A recent next-generation sequencing study on 11 SNUC revealed frequent gains at 1q, 3q, 8q and 17q and losses at 3p and 17p^27^. These regions also stood out in our data. We were unable to find genetic studies on SNEC in the literature.

SNUC, even when defined as Ck+Ne−/SNUC, may still be considered a heterogeneous group of malignancies. The 2017 edition of the WHO classification of head and neck tumours recognizes NUT carcinoma as a separate entity related to the group of midline carcinomas that generally are highly aggressive, while with regard to SMARCB1-deficient carcinoma it remains unclear wether it constitutes a distinct entity^1^. Additional immunohistochemical analysis of our Ck+Ne−/SNUC cases yielded one case with partial NUT expression, however, this was below the threshold of positivity^28^ and therefore not considered a true NUT carcinoma. Moreover, this tumour demonstrated multiple chromosomal segments involved in gains and losses (Fig. 4), while NUT carcinomas typically have simple karyotypes often with the t(15;19) translocation as sole aberration^18^. We have no explanation for the NUT expression in this tumour, normally it is restricted to the germ cells of the testis and oocytes. Two Ck+Ne−/SNUC cases showed complete absence of SMARCB1 staining and both had simple karyotypes, which is in agreement with the literature^29^. One of the two SMARCB1-deficient cases carried a homozygous deletion on 22q11, where the SMARCB1 gene is localized (Fig. 3), indicating that both copies of the gene had been lost. On the basis of these results and the recommendations of the WHO^1^, we decided to not separate these three tumours from our Ck+Ne−/SNUC group.

Correct classification of tumours, either by histological examination, immunohistochemical staining or genetic analysis, is important for prognosis and therapeutic decision-making. For a growing number of sinonasal tumour types, genetic profiling is the defining tool for classification. Examples are characterizing chromosomal translocations t(15;19) NUT-BRD4 in NUT carcinoma, t(11;22) EWSR1-FLI1 in sinonasal Ewing sarcoma/PNET and chromosomal rearrangements involving PAX3 Biphenotypic sinonasal sarcoma^15–18^. Further, HPV-related carcinoma with adenoid cystic-like features (also named HPV-related multiphenotypic sinonasal carcinoma) is defined by the presence of high-risk HPV in the absence of the t(6;9) MYB-NFIB rearrangement^15–17,30^. Recently, also tumour-specific mutations have been reported, such as EGFR exon 20 mutations in 88% inverted papilloma and in 77% of squamous cell carcinoma associated with inverted papilloma, and KRAS mutations in oncocytic papillomas^31,32^. Perhaps the recently described IDH2 mutations may define a subset of SNUC^27,33^. Apart from the analysis on possible cases of SMARCB1-deficient carcinoma and NUT carcinoma, in this study we did not test for these additional characterizing genetic abnormalities; although we analysed copy number alterations on a genome-wide scale, our method was limited by the fact that characterizing mutations were not evaluated. Clearly there is a need for further genetic studies using next-generation sequencing.

As the low incidence of these rare tumours hampers the clinical testing of modern therapies targeted to these specific genetic aberrations, multicenter studies and basket trials will be mandatory. Bromodomain inhibitors that provoke squamous differentiation and inhibit cell proliferation may be used for patients with NUT carcinoma, and the first clinical results of appear promising^34^. Second- and third-generation EGFR inhibitors are being developed and tested for lung cancer patients and may be effective also for sinonasal tumour with EGFR exon 20 mutations^31^.

In conclusion, copy number alteration profiles of three poorly differentiated and difficult to diagnose sinonasal tumours ONB, SNEC and SNUC were more distinctive of each tumour type when classified by immunohistochemical staining with cytokeratin and neuroendocrine markers. As immunohistochemistry is routinely used in pathology, our genetic data support the usefulness of this simple staining in the diagnosis of these tumours, which in turn may aid prognostication and therapeutic decision-making.

## Material and Methods

### Patients and tumour material

This study included a total of 54 patients diagnosed as ONB, SNEC and SNUC between 1988 and 2011 from Hospital Universitario Central de Asturias (HUCA), Oviedo Spain, from VU Medical Center (VUmc), Amsterdam, The Netherlands and from University Medical Center Utrecht (UMC), Utrecht, The Netherlands. All experimental protocols were approved by the Institutional Ethics Committee of the Hospital Universitario Central de Asturias and by the Regional CEIC from Principado de Asturias (approval number: 66/15 for the project PI15/01629). All methods were carried out in accordance with the guidelines of the Institutional Ethics Committee of the Hospital Universitario Central de Asturias. Informed consent was obtained from all patients. All cases were classified according the criteria of each anatomic pathology service that indicated 24 cases as ONB, 11 as SNEC and 19 as SNUC. According to the 2017 WHO histological classification^1^, 13 cases ONB cases were low-grade (I–II) and 11 were high-grade (III–IV). SNEC and SNUC cases were not histologically further subclassified (Table 1).

### Immunohistochemistry

Tissue microarray (TMA) blocks were prepared from formalin fixed, paraffin embedded tumour tissues using the Beecher Tissue Microarray (Beecher Instruments, Silver Spring, MD, USA). In total 4 TMA blocks were constructed, containing three 1 mm cores from different areas of 54 tumours. Each block included normal sinonasal mucosa samples as internal control. Three micrometer sections were stained with hematoxylin and eosin and reviewed by one pathologist to determine whether the samples contained a good representation of the original tumour blocks. Immunohistochemistry was performed on an automatic staining workstation (Dako Autostainer Plus; DakoCytomation, Glostrup, Denmark) with antigen retrieval by EnVision FLEX + Mouse (DakoCytomation, Glostrup, Denmark) during 20 minutes. The following antibodies were applied: prediluted mouse anti-Cytokeratin clone AE1/AE3, mouse anti-Synaptophysin clone SY38 and mouse anti-Chromogranin A clone DAK-A3 (DAKO, Glostrup, Denmark), 1:80 diluted mouse anti-INI-1 clone MRQ2 7/ZSI1 (Gennova, Sevilla, Spain), and 1:45 diluted rabbit anti-NUT-1 clone C52B1. The slides were evaluated in a double-blind manner by three observers (ALH, BV and MAH). Positivity for Ck was considered when >10% tumour cells showed membranous/cytoplasmic staining; Ne was evaluated as positive when either or both synaptophysin or chromogranin showed membranous/cytoplasmic staining in >10% tumour cells. ONB was defined as Ck−Ne+, SNEC as Ck+Ne+ and SNUC as Ck+Ne−. NUT-1 was scored as positive when >50% of tumour cells showed nuclear/cytoplasmic staining. Loss of SMARCB1 staining was defined as complete absence of staining on tumour cells while positivity is observed on stromal or inflammatory cells.

### DNA extraction and genome-wide copy number analysis

The DNA was obtained from 10 slides of 3 µm of FFPE tissue, after identification of tumour-enriched regions (BV) in a section stained with Haematoxylin and Eosin. Special care was taken to obtain high-quality DNA from the formaldehyde-fixed, paraffin-embedded tissues. We applied an elaborate extraction protocol^35^ which includes thorough deparaffinization with xylene, methanol washings to remove all traces of the xylene, and incubation in sodium thiocyanate to reduce cross-links. Subsequently, the tissue pellet was digested for 3 days in lysis buffer with high doses of proteinase K freshly added twice daily. Finally, DNA extraction was done using the ROCHE high pure PCR template preparation kit (Roche Diagnosis GmbH, Mannheim, Germany) according to the manufacturer’s recommendations. Quantification of DNA was performed using Quant-iT™ PicoGreen®dsDNA Assay Kit (Thermo Fisher Scientific, Madrid, Spain) following the manufacturer’s protocol.

Genome-wide copy number analysis was performed using the OncoScan FFPE Assay Kit (Affymetrix, Santa Clara, CA, USA) according to the manufacturer’s recommendations. All tumour samples were batch-normalized against internal Affymetrix controls. Copy number alterations (CNA) were analysed with Nexus Express for OncoScan 3.0.1 (BioDiscovery, Hawthorne CA, USA). At least ten probes per segment were considered as the minimum number to define a copy number alteration. Gains were called if the log2 ratio was >0.2 and losses <−0.2. High copy number gains were scored when the log2 ratio >1.2 and homozygous deletions <−1.2.

### Data availability statement

All data generated in this study will be made available to those interested.
